# Supplementary material for: Sustainable and Active Program—Development and Application of SAVING Methodology
Source: Int J Environ Res Public Health. 2022 Jun 2;19(11):6803. doi: 10.3390/ijerph19116803 (PMC9180391; doi:10.3390/ijerph19116803)
Supplement: Supplementary file 1 [file ijerph-19-06803-s001.zip › ijerph-1727946-supplementary.pdf]

International Journal of
Environmental Research and Public Health

MDPI

Supplementary Material

The Supplementary Material, it is presenting the details regarding each theme to calculate the SAVING Index.

S1. SAVING INDEX—Administrative Data, Physical characteristics, and Building usage

Table S1. Administrative Data, Physical characteristics, and Building usage.

**Administrative Data**	
Elderly Home Name	
Type of Elderly Home (Home Support/Day Center)	
Age Group of Users	
Country	
City	
Address	
Name/Function (persons who provided information for this questionnaire)	
**B) Physical characteristics**	
**Construction year**	
Elderly Home Area (m2) (Total area that includes outdoor area: patios, gardens, ...)	
Area with Buildings (m2)	
Usable surface area (m2)	
Closed conditioned area (m2)	
Number of Floors (includes underground)	
Number of Rooms	
Area of the Rooms (average value in m2)	
Number of Bedrooms	
Bedroom Area (average value in m2)	
Canteen (Y/N)	
Gym (Y/N)	
Description and year of any heating, ventilation and air conditioning and refrigeration (HVAC&R) system remodeling or upgrade process	
**C) Building usage**	
**Number of Occupants**	
Users	
Sociocultural Animator/Social Worker/Psychotrician	
Nurse	
Direct Action Helper	
Direct Action Helper with a view to reinforcement at night	

In charge of Domestic Services	
Helper	
**Number of occupants per Room (average value)**	
**Number of Cantina users/Day (average value)**	
**Number of Gym users/Day (average value)**	
**Observations/comments on the use of the Building**	

S2—SAVING INDEX - Energy consumption, Energy production, Lighting, Heating, Cooling, Ventilation, Energy measurement, Power measurement, Energy audits, and Building features

Table S2. Energy consumption, Energy production, Lighting, Heating, Cooling, Ventilation, Energy measurement, Power measurement, Energy audits, and Building features.

**D) Energy consumption**	
**Is electricity consumed? (Y/N))**	
Average annual bill (kWh, €)	
What are the main uses of electricity (eg air conditioning, ventilation, lighting, ...)?	
**Is natural gas consumed? (Y/N)**	
Average annual bill (kWh, m3, €)	
What are the main uses of natural gas (eg hot water, heating, ...)?	
**Is propane/LPG gas consumed? (Y/N)**	
Average annual invoice (Kg, €)	
What are the main uses of propane/LPG gas consumed? (eg hot water, heating, ...)?	
**Is gasoline/diesel consumed? (Y/N)**	
Average annual invoice (Kg, €)	
What are the main uses of gasoline/diesel (eg hot water, heating, ...)?	
**Are other fuels or other energies consumed? (Y/N) Which one? (eg: biomass)**	
Average annual bill (energy unit, €)	
What are the main uses of the other fuels or other energies used (eg hot water, heating, ...)?	
**Observations/comments on energy consumption**	
**E) Energy production**	
**Is there renewable energy production (eg photovoltaic panels)?**	
What part of this energy is consumed in the building?	
**Observations/comments on energy production**	
**F) Lighting**	
**Interior lighting**	

What type of lighting is predominantly used in the building (eg fluorescent, incandescent, halogen, LED, ...)?	
What are the opening hours of the interior lighting?	
Are there systems for automatic control (eg motion sensor, time clock, photocell, ...)? Which ones and where?	
**Outdoor lighting**	
What type of lighting is predominantly used outdoors (eg fluorescent, incandescent, halogen, LED, metal halide...)?	
What are the opening hours of the outdoor lighting?	
Are there systems for automatic control (eg motion sensor, time clock, photocell, ...)? Which ones and where?	
**Observations/comments on lighting**	
**G) Heating**	
**Are there heating systems in the building?**	
**Equipment used for heating in the building:**	
System type (centralized system, individual units, ...)?	
What is the temperature value set on the heating equipment?	
Type of equipment (eg heat pump, boiler, radiators...)	
Type of energy/fuel consumed (eg electricity, natural gas, propane, ...)	
Approximate year of system installation	
System make and model (most common system)	
**Hours of use**	
The heating is used in which months of the year?	
How many hours a day is the heating used during these months?	
**Are there regular maintenance works?**	
Maintenance frequency definition	
Definition of annual maintenance cost	
**Observations/Comments on Heating**	
**H) Cooling**	
Are there cooling systems in the building?	
Equipment used for cooling in the building:	
System type (centralized system, individual units, ...)?	
What is the temperature value set on the heating equipment?	
Type of equipment (eg: air conditioning, heaters...)?	
Type of energy/fuel consumed (eg: electricity, natural gas, propane, ...)	
Approximate year of system installation	
Brand and model of the system	
**Hours of use**	
The cooling is used in which months of the year?	

How many hours a day is cooling used during these months?	
**Are there regular maintenance works?**	
Maintenance frequency definition	
Definition of annual maintenance cost	
**Observations/Comments on Cooling**	
**I) Ventilation**	
**Is there mechanical ventilation in the building?**	
Type of ventilation system/equipment	
Approximate year of system installation	
How many hours a day is mechanical ventilation turned on in the building?	
**Is there natural ventilation in the building?**	
Type of natural ventilation system/equipment (eg windows, free-cooling system,...)	
Does the ventilation operate at night?	
**Is there mechanical ventilation in other areas? At where? (Ex.: canteen, bathroom, bedrooms, ...)**	
Type of ventilation system/equipment	
Approximate year of system installation	
How many hours a day is the ventilation system operating?	
**Is there natural ventilation in other areas (eg canteen, bathroom, ...)? At where?**	
**Is there regular maintenance work (mechanical ventilation)?**	
Definition of maintenance frequency per system	
Definition of annual maintenance cost per system	
**Observations/Comments on Ventilation**	
**K) Medição de Energia**	
**Energy measurement (Y/N and where)**	
Power distribution table?	
Partial distribution tables?	
**Natural gas measurement (Y/N and where)**	
General accountant?	
Partial counter?	
**Propane/LPG metering (Y/N and where)**	
General accountant?	
Partial counter?	
Observations/Comments on Energy Measurement	
**L) Power management**	
**Identification of the entity/person responsible for Energy Management**	

The role, tasks, and main responsibilities?	
Are heating, cooling, and ventilation systems operated manually or automatically?	
**Is there an energy management system?**	
**Observations/Comments on Energy Management**	
**M) Energy Audits**	
**Has any energy audit been carried out in the building before?**	
Audit date	
**Observations/Comments on Energy Audits**	
**N) Building Features**	
**How do you assess the quality of the building's facade and roof?**	
Definition of facade and roof layers (if data is available)	
Average facade and roof thickness (e.g. wall thickness, measured through a window or opening)	
Are there leaks on the building's facade? At where?	
**How do you rate the quality of the building's windows?**	
Definition of windows (eg: sliding or hinged)	
Are there leaks through the windows? At where?	
Characterization of glass and window frames	
**Shading devices**	
Are there any external shading elements (eg curtains, blinds, etc.)?	
Are there any interior shading elements (eg blinds, curtains, etc.)?	
Are there natural or architectural shading elements of the building (eg trees, building elements,)?	
**Observations/Comments on the Building**	

S3—SAVING INDEX - Comfort

Table S3. Comfort

**Comfort**	
How do you evaluate the thermal comfort felt in the building (high/medium/low)?	
How do you rate the visual comfort felt in the building (high/medium/low)?	
How do you classify the outside noise in the building (high/medium/low)?	
**Comments/Comments on Comfort**	
(eg: are there areas of the building that are too cold in winter or too hot in summer, ...)?<br>(eg, are there areas of the building with poor or very bright lighting)?	

S4—SAVING INDEX - Indoor air quality

Table S4. Indoor air quality

Indoor air quality	
Is the Home located near a street with low, medium, or high traffic?	
Is there a space in the home where smoking is allowed?	
-If yes, where?	
Select 2 interior spaces where the QAI will be developed and characterize each of them	
Localization	
Name	
-area (m2)	
-height (m)	
-number of users	
-floor type	
-type of windows	
Electricity available (Y/N).	
-Number and location of electrical plugs.	
**Observations/Comments on QAI**	

S5—SAVING INDEX - Waste management

Table S5. Waste management

Waste management		
Is there waste separation for recycling?		
Which ones?	Paper	
	Plastic	
	Metals/Cans	
	Glass	
	WEEE (Waste Electrical and Electronic Equipment)	
	Others (batteries, corks, ...)	
**Waste Accounting**		
Is there any accounting for the amount of waste produced?		
Is there any accounting for the amount of waste sent for recycling?		
How this accounting is done		
**Reuse of Waste**		
Is the paper reused?		
**Observações/comentários sobre Gestão de Resíduos**		

S6—SAVING INDEX - Water

Table S6. Water

Water		
Average monthly invoice (m3, €)		
Are there devices to save water consumption?		
Which ones?	Flow faucets	
	Dual sanitary flush system	
	Other (indicate which one)	

**Sanitary Hot Waters (DHW)**		
Is there hot water consumption in the building?		
What kind of equipment is used to produce hot water?	Heat pump	
	Boiler	
	Cylinder	
	Other (indicate which one)	
What is the temperature value for hot water (eg in the storage tank)?		
What kind of energy/fuel is consumed?	Electricity	
	Natural gas	
	propane gas	
	Other (indicate which one)	
Approximate year of system installation		
Are there solar thermal collectors installed and in operation?		
How many thermal collectors are installed and in operation?		
Hot water is used in which months of the year?		
Where is hot water used?	Kitchen	
	bathrooms	
	baths	
	Other (indicate which one)	
**Water sources**		
Available water sources:	Public Network Supply	
	Pit	
	Hole	
	Other (indicate which one)	
**Observations/Comments on Water**		

e.g. Make and model of DHW system (most common system)

S7—SAVING INDEX - Mobility

Table S7. Mobility

**Mobility**	
**Car Parks**	
Number. of parking spaces in the Home or outskirts (up to a radius of 100m)	
On one. of parking spaces for the disabled in the Home or outskirts (up to a radius of 100m)	
On one. of parking spaces for trams in the Home or outskirts (up to a radius of 100m)	
On one. of parking spaces for bicycles in the Home or outskirts (up to a radius of 100m)	
Photo of the car park	
**Characterization of the Transport Network**	
**Bus**	
Number of stops within a radius of 500m	
Average daily frequency of passage per hour	

Distance between the nearest stop and Home	
**Metro/Subway**	
Number of stops within a radius of 500m	
Average daily frequency of passage per hour	
Distance between the nearest stop and Home	
**Train**	
Number of stops within a radius of 500m	
Average daily frequency of passage per hour	
Distance between the nearest stop and Home	
**Electric/Tram**	
Number of stops within a radius of 500m	
Average daily frequency of passage per hour	
Distance between the nearest stop and Home	
**Boat**	
Number of stops within a radius of 500m	
Average daily frequency of passage per hour	
Distance between the nearest stop and Home	
**Observations/Comments on Transport**	

S8—SAVING INDEX – Green spaces

Table S8. Green spaces

General information	
Total garden area (m2)	
The total lawn area (m2)	
Suitable for use by users?<br>Existence of public gardens nearby (max. 250m from the Home). If yes, what is your area?	
**Energy**	
Type of fuel used in gardening activities	
Annual diesel consumption in gardening activities (l/year, €)	
Annual gasoline consumption in gardening activities (l/year, €)	
Annual consumption of agricultural diesel in gardening activities (l/year, €)	
Annual electricity consumption in gardening activities (kWh/year, €)	
**Water**	
Type of irrigation system	
-Origin of irrigation water	
Water consumed for irrigation (m3/year, €)	
**Gardening Treatments**	
Name of each pesticide used	
Amount used of each pesticide (Kg/year)	
Name of each fertilizer used	
The used amount of each fertilizer (Kg/year)	
Type/Name of compost used (is it done as home dressing?)	
Amount of compost used (kg/year)	

**Biome information**	
number of trees	
Predominant tree species	
The average age of trees	
**Observations/Comments on Green Spaces**	

S9—SAVING INDEX - Activity and Organization

Table S9. Activity and Organization

Active Ageing Events	
Is there a formal program of cultural and leisure activities?	
- Diversity of activities developed (Reading activities – a type of reading – the quality of readings, conversations, discussion of topics, games, gardening, etc.)	
- Number of activities developed/year (by scales: 0; 1-3; 4-10; more than 10)	
-Number of cultural outings/year (by scale: 0; 1-3; 4-10; "more than 10"/week or month)	
How do the activities suit the characteristics of the users?	
How do the activities suit the needs of users?	
Periodic variation of activities and respective program (weekly, monthly, six-monthly, annual)	
Are there tours abroad?	
-How often are the tours?	
-How diverse are the tours? (garden, etc.)	
-How long are the tours? (eg for 1h)	
**Relationship between professionals and users/family members**	
Information system for family members of users	
-Relationship with users' relatives (visit times)	
Is there a complaints system?	
-Is there a possibility of verbal complaints?	
-Is there a suggestion box?	
What is the relationship between employees and users? (sympathy, attention; interest; empathy; respect)	
**Internal organization and Human Resources of the Center**	
**Is there an internal evaluation system?**	
- Is the system coherent?	
**Is there an external evaluation system?**	
-Is the system coherent?	
**Is there an employee performance appraisal system? If yes, which one?**	
-Is the system coherent?	
**Is there an activity report?**	
**Is there an activity plan?**	
**Staff table**	

-What are the qualifications of the employees?	
-What is the contractual status of employees?	
-How long are the employees?	
-What is employee turnover?	
**Are there training actions for employees?**	
-What is the frequency of training actions?	
-What is the scope of the training actions?	
-What is the adequacy of training actions?	
**Observations/Comments on Activities and Organization**	

S10—SAVING INDEX – Green deal

Table S10. Green deal

**Certification information**	
ISO 14001: 2004 Certificate - Environmental Management Systems, taking into account environmental protection, pollution prevention, legal compliance, and socio-economic needs or any other certification related to the environment (Y/N)	
Policies, objectives, or goals for the conservation of the environment (Y/N)	
Number of individuals in the Home trained in "green commitment"	
Number of individuals with an "eco-driving" certificate	
**Electronic equipment information**	
**Energy Efficiency Label**	
-Number of equipment with A+++ (A)	
-Number of devices with A++ (B)	
-Number of equipment with A+ (C)	
-Number of equipment with A (D)	
-Number of equipment with B (E)	
-Number of equipment with C (F)	
-Number of equipment with D (G)	
-Number of equipment without energy efficiency label	
**V2.2 Printers**	
-Number of printers	
-Number of printers with consumption optimization	
-Amount of paper used (Kg/month)	
-Amount of paper purchased directly from domestic producers (Kg/month)	
-Amount of recycled paper used (Kg/month)	
-Use of chlorine-free paper? (Y/N)	
**Chemicals**	
Concern about chemical information on labels of detergents and other products (Y/N)	
**Food products information**	
The total amount of food purchased per month (kg/month)	
The total amount of food purchased with organic certification per month (Kg/month)	
Place of purchase of food products	

-Num. from local suppliers	
-Num. from district suppliers	
-Num. from national suppliers	
-Num. from local international suppliers	
**Observations/Comments on Green Commitment**	

S11—SAVING INDEX – Physical activity and Exercises

Table S71. Physical activity and Exercises

**Physical activity**	
Activity/Movement/Exercise Programs	
Average hours/week of movement/physical activity	
**Health and Wellness Promotion**	
Intergenerational programs	
Outreach programs (eg lectures, outreach, …)	
If yes, how many per year?	
Possibility of therapeutic movement	
Availability of Physiotherapist/occupational therapist/speech therapist/other professional	
Interior route without obstacles	
Outdoor route without obstacles	
Exterior paths for movement (in addition to the single gait of W2.6)	
Slope courses	
Routes with stairs	
**Observations/Comments on Physical Activity - Therapeutic Movement**	
